# Alpine life is shaped by topography

**DOI:** 10.1007/s00035-026-00360-8

**Published:** 2026-06-16

**Authors:** Christian Körner, Erika Hiltbrunner

**Affiliations:** Department of Environmental Sciences, University of Basel, Schönbeinstrasse 6, Basel, CH-4056 Switzerland

**Keywords:** Alpine ecosystems, Biodiversity, Climate, Exposure, Flora, Microclimate, Nunatak, Snowbed, Relief, Ruggedness

## Abstract

Aspect and steepness with ridges and depressions of different spatial extent, hence, the topography of high-elevation terrain affects the incidence angle of sun rays, the influence of wind, the redistribution of snow and the sorting action of gravity for water, nutrients, diaspores and sediments. These factors jointly shape life in the treeless alpine world, beyond the influences related to the strong vertical profile of the air temperature with a mean lapse rate of 0.55 K 100 m^−1^. On top of diverse soil types, the resulting thermal habitat conditions within a given elevation belt substantially exceed the current (+2.9 K) and future (+4 K) climatic warming in the European Alps. To account for the full spectrum of life conditions, the spatial scale of analysis is essential. We show that pronounced topography effects on microclimate on nunatak-type cliffs above the trimline of glacial ice shields could most likely not fully counteract the then lower and hostile temperatures. Life conditions across snowbeds exemplify the effect of season length on alpine plant’s phenology and growth over short distances, with halved aboveground biomass production for a shortening of the season by two to three weeks. Furthermore, we highlight potential pitfalls in microclimate assessments of topography effects in open terrain, but also explain how to improve such measurements. Throughout this review, we emphasize the interactive effect of plant canopy structure (height, density) with topography, mutually creating life conditions not predictable from standard meteorological sources, but responsible for the over-proportional plant species richness of the alpine flora globally.

**Supplementary Information:**

The online version contains supplementary material available at https://doi.org/10.1007/s00035-026-00360-8.

## Introduction

Whenever land becomes elevated, tectonic forces and the action of gravity interact, shaping the land surface into contrasting aspects, steepness, and roughness. Past glaciation or the glacial-interglacial cycles have profoundly reshaped the land surface by acting as primary agent of erosion and through massive landscape modification. The resulting topography interferes with solar radiation, wind, precipitation, bedrock and substrate. The outcomes are myriads of life conditions at small scale that cause differences in soils and plant cover, which in turn feed back on the microenvironment. The resulting small-scale patterns of microclimatic and soil conditions host biota of divergent habitat preferences at rather short distances, causing the alpine life zone to be richer in species than expected from its land area (Körner 2004, 2021; Praeg et al. 2025).

Soils, similar to plants, are affected by the parent rock material, sediment allocation, input and drainage of water, snow duration, and the temperature regime, and they develop in close concert with plant communities, particularly during initial stages (Burga et al. 2010; for the Arctic alpine: Górniak et al. 2017). The resulting successional cycles not only include plants and soils, but also the soil biota (Rime et al. 2015). Soil formation usually continues for thousands of years, initiated by small patches of substrate and few pioneer plant species. Remarkably, the specific bedrock chemistry (calcareous vs. acidic) facilitated or restricted the plant species’ migration paths during the glacial and interglacial periods (Schönswetter et al. 2005; Alvarez et al. 2009).

It had long been noted that aspect and relief are key drivers of plant distribution in the alpine terrain above the climatic treeline (Schröter 1926; Billings and Bliss 1959; Aulitzky 1961, 1963; Ellenberg 1963; Geiger 1965; Kirkpatrick and Bridle 1999; Löffler et al. 2006; for more recent literature see Körner 2021; Oldfather et al. 2024). Reading these classical texts on topography effects on plants, reminds one of a ‘re-invention of the wheel’ in recent years, and a need to make these early works available in English. For instance, Fritz Kerner (1893, 1917; referenced in Schröter 1926) monitored the highly buffered, monthly soil temperatures at 80 cm depth at eight compass directions in open montane terrain for a number of years, documenting a 2.2 K North-South range of means that corresponds to a 400 m difference in elevation. In the summit region at 2300–2600 m a.s.l., he reported 4–5 K difference in annual maxima in 30 cm soil depth. Such exposure contrasts can reach 40 K in the top 1 cm under bare soils in a diurnal course (Aulitzky 1961). A modern analysis of aspect effects on high mountain summits and different slope exposure revealed clear cut consequences for plant species and plant functional type distribution (Winkler et al. 2016; Thakur and Chawla 2025, respectively).

While exposure to the sun is an obvious factor determining the radiation balance, which affects all energy driven processes (e.g., Plüss and Ohmura 1997), topography exerts many other influences at a small scale as mentioned above. Wind exposure or shelter influence aeolic sedimentation, but also the accumulation and melting regime of snow. Depressions and gullies are channelling and affecting retention of water, the transport of substrate, organic debris, and nutrients, and the steepness also adds a mechanical force to the plants. The impact of wind and topography has already been quantified almost exhaustively by Aulitzky (1963) in an attempt at identifying ‘safe sites’ for seedlings. Topography also causes differences in the degree of aerodynamic coupling of low stature vegetation (humidity and temperature among plants). And vice versa, plants can enhance the aerodynamic decoupling from the free atmosphere by their canopy height and density. Certain topographic features select for specific life forms and their shape, a process that increases or compensates the pure topography effect (e.g., in the case of wind-edge plants). Consequently, there are strong plant stature-topography interactions. Correspondingly, topography influences soil quality and soil depth and thereby co-determines the below- as well as the aboveground plant structures. The net-outcome is a mosaic of microhabitats that select for different types of plant life forms, plant species and plant communities.

The classification of different plant life forms by the position and protection of buds, dates back to 1930s (Raunkiær 1934) and has repeatedly been applied to different, local floras, including the alpine (Mark et al. 2000; Midolo et al. 2024). Perennial plants are usually inactive during hostile periods (frost, drought), while their buds need to be protected in order to guarantee the formation of leaves and flowers on time, after the end of the adverse season. These traits become selected for by topography, either by providing exposure or shelter.

Since those early days of topography-related microclimatology, the advance in technology has permitted refining and expanding data collection, most importantly, by data logging (mainly temperature [T]) and by employing thermal imagery. The examples presented in this review will capitalize on these more modern tools. We have organised our review first into three sections along the «axes of space and time» in order to distil the relevant scaling for an understanding of alpine plant species, their distributions and their expected responses to environmental changes. Finally, we offer some guidance toward an unbiased assessment of temperatures across topographic gradients.

### When topography matters, spatial scale matter as well

At a 100 km^2^ or larger grid, as often employed for biodiversity inventories, (1) entire mountain floras –from foot hills to summits– fall into one grid cell, explaining why mountains pop up as hotspots of biodiversity (e.g., Mutke and Barthlott 2005), given they assemble climatic zones that otherwise would latitudinally spread over thousands of kilometres. At the same time, this is the best example for the overarching role of topography for the diversity of life at a very large, global scale. At this coarse scale, it is impossible to associate the occurrence of species or plant communities with environmental drivers other than perhaps latitude (affecting season length), mountain size (maximum elevation, range of climatic belts included) and distance from oceans (oceanity, mass elevation effect). Once defined, the mountainous regions of the world can be inventoried (Körner et al. 2017; Snethlage et al. 2022).

At the spatial resolution of summits, ridges or valleys, gullies and depressions, (2) an intermediate scale encompasses topographic shelter, wind- and leeward exposure, snow cover duration, temperature inversion, cold air and water drainage, avalanche tracks and debris flows, all shaping habitat conditions at the scale of a few meters up to several hundred metres. At that mesoscale (Billings 1973; Oldfather et al. 2024), the alpine terrain becomes fragmented into diverse types of edaphic and microclimatic conditions at otherwise similar macro-climate. The mesoscale topographic diversity leads to what is often addressed as β-diversity, that is, the diversity of plant communities. High-resolution maps of plant assemblages, as the ones collected for the Alps (Ozenda 1963–1988; Ozenda and Borel 2003), could gain novel power, when combined with GIS and further spatially relevant ecological information. Also termed as geodiversity, this topographic diversity at the mesoscale is one of the reasons for the over-proportional species diversity in alpine terrain (Körner 2004; Körner and Hiltbrunner 2021; Oldfather et al. 2024; Praeg et al. 2025). A good example of plant distribution modelling at this mesoscale was elaborated for a rock-inhabiting, rare *Saxifraga* species in order to identify its likely micro-refugia in the Maritime Alps, covering a presumable presence in this region of 21’000 years (Patsiou et al. 2014).

In stark contrast, the environment experienced by individual alpine plants, is determined (3) at a scale of less than 1 m^2^, often at the scale of a few cm. In fact, alpine plants ‘engineer’ their microclimate by their stature (an extreme case are cushion plants), with the differences to concurrent air temperature at 2 m above ground exceeding 20 K on a bright day (Cernusca 1976; see Fig. 4.8 in Körner 2021). Hence, any attempt at explaining alpine plant distribution by wind, temperature, moisture, snow cover, etc. requires data at that spatial scale. With regard to climatic data, this scale is commonly addressed as ‘microclimate’ by plant and animal ecologist (Bramer et al. 2018; Körner and Hiltbrunner 2021), although the same term is also applied to ‘local’ climate by climatologists (e.g., the ‘microclimate’ of a valley, see Pepin et al. 2025), in contrast to ‘macroclimate’ for larger regions, which causes confusion, unless defined.

The thermal conditions at the micro-topography scale reflect the physics involved, which determine the deviations from the free atmosphere, largely independent of elevation. Thus, in an alpine environment, shifts in atmospheric temperatures (e.g., with altitude) will cause similar shifts in microhabitat temperatures, while still retaining the contrasts among microhabitats as long as no other environmental change interferes (e.g., shifts in drainage and sedimentation or snow distribution). In a first approximation, proportional shifts will occur across microhabitats under continuing climate warming. As mountains narrow with elevation, some microhabitat types will decline and others will gain in abundance (Scherrer and Körner 2011). But as long as mountains are high enough, the thermal microhabitat types will not get fully lost (Körner and Hiltbrunner 2021).

The climatic drivers for the life form «tree» operate (4) much less topography-dependent, because trees (with a height of at least 2–3 m) are aerodynamically strongly coupled to ambient air temperature. It is the individual tree crown that interacts with solar radiation rather than the entire forested slope, hence, similar temperatures and treeline positions on N- and S-slopes in the Alps were monitored (Paulsen and Körner 2001; Körner and Paulsen 2004; Körner 2012). Topography effects on mountain forests may play a role through avalanche tracks, rockfall, gully erosion, mudflows, water logging and limited water availability on outcrops, as well as topography-guided routes of fire-spreading. A classical, topography-related phenomenon is the depression of the upper tree limits in gulleys and their advances on ridges near the temperate and boreal alpine treeline due to avalanches and late winter snow duration, but the opposite pattern has been observed in the tropics (Körner 2012). These topography driven patterns of uppermost tree distribution may also shift the alpine flora downslope, locally, thus, cold-air drainage may affect both (Körner 2012).

The position of the undisturbed, steady-state treeline matches the 6 °C isotherm of seasonal mean air temperature globally, embedded in the vertical temperature profile of the atmosphere (mean lapse rate of 0.55 K 100 m^−1^ with seasonal variation, Kollas et al. 2014). It serves as a reference line for the bioclimatic belts in mountains, termed colline, montane, alpine and nival belts, with the treeline separating the alpine from the montane world, irrespective of whether trees are locally absent because of disturbances (Körner et al. 2011; Körner and Hoch 2023). The term 'subalpine' is still in use in some regions and refers to the uppermost montane forest or its substitute vegetation. These elevation belts can be associated with certain thermal regimes, both in high (Körner et al. 2011) and low latitude (Winiger 1981) mountains. At this scale, weather station data (or proxies thereof obtained from shallow, fully shaded soil by temperature loggers) are adequate to define the position of these belts at and below treeline (e.g., Körner and Paulsen 2004; Körner et al. 2011, 2017; Paulsen and Körner 2014).

Returning to the relevant scale for the alpine flora above treeline, we refer to an analysis by Scherrer et al. (2010, 2011). They used data from a large data logging campaign (173 T-loggers) combined with thermal imagery across topographically rich alpine and arctic terrain to explore the scale dependency of a 2–4 K climate warming scenario on alpine plant species distribution. Accounting for plant temperatures, the projections at a 1 m resolution and a 100 m resolution differed enormously: A 2 K warming caused an 8% vs. 65% loss of habitats at a 1 vs. 100 m scale, whereas a 4 K warming a 40% vs. 100% loss (Scherrer et al. 2011). In other words, almost all species-specific thermal habitats would disappear at a 100 m scale, while 60% of the habitats were retained at a 1 m scale, causing Körner and Hiltbrunner (2021) to emphasize a particular robustness of the alpine flora to such climatic warming scenarios, owing to microtopography. This persistence of species across larger regions also roots in the diverse microhabitats occupied by different subpopulations within species (Oldfather and Ackerly 2019). Importantly, by ‘flora’, we refer to the plant species inventory of a region (regions at which floras are defined, such as the Swiss Alps or the Scandes; e.g., Snethlage et al. 2022), and not to the potential effects on a given plant in its current habitat (e.g., a certain summit). The local differences between air temperature (standardized at 2 m above ground) and the temperatures plants actually experienced, exceeds by far any climate warming scenario (Scherrer and Körner 2011; Graham et al. 2012). Hence, the thermal microhabitat diversity is key for the diversity of the alpine flora (e.g., Körner 2004; Vittoz et al. 2010; Opedal et al. 2015).

**Fig. 1:**
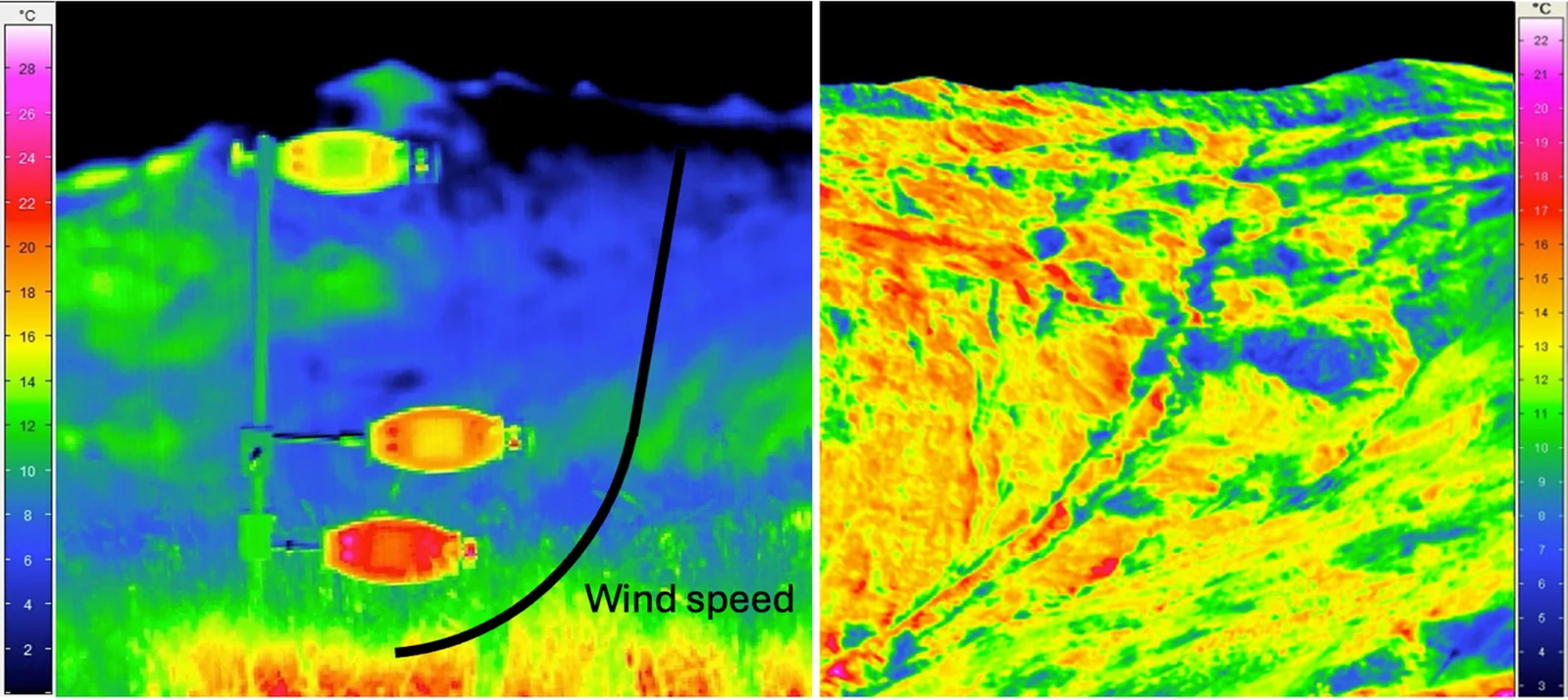
Left: Wind modifies the convective heat transfer as visualized by three inert bodies (in this case small anemometers) installed at different heights above alpine grassland: the closer to the ground, the higher the temperature (the less convective heat transfer) under otherwise identical solar radiation. The lowest box slightly exceeds the temperature of ground vegetation beneath (by c. 2–4 K) due to plants’ latent heat loss (transpiration). Right: Thermal patchiness follows topographical patchiness, surface temperature varies between 5 to 22 °C on an alpine slope, centred around 2500 m elevation (from Scherrer and Körner 2010)

In addition, there is also a vertical microscale at which topography and plant stature interact: plants on convex terrain (ridges, humps) are small, while on concave ground (hollows, gulleys) they are often taller, with the exception of the centre of high elevation snowbeds where only small plants can handle the extremely short growing season. These differences in stature are both phenotypically (plant height adjustments; e.g., Takahashi and Yoshida 2009; McIntire et al. 2016) and genotypically controlled (selection of species; e.g., ‘wind-edge species’ in Bronken Eidesen et al. 2013). Different plant morphologies affect heat convection in and above plant communities. Since these relationships are not visible, thermal imagery can help: a dry, inert body exposed at different heights (and thus wind speed) above ground, will depart from standard air temperature as a consequence of the interaction between solar radiation and convective heat exchange (Fig. 1).

**Fig. 2:**
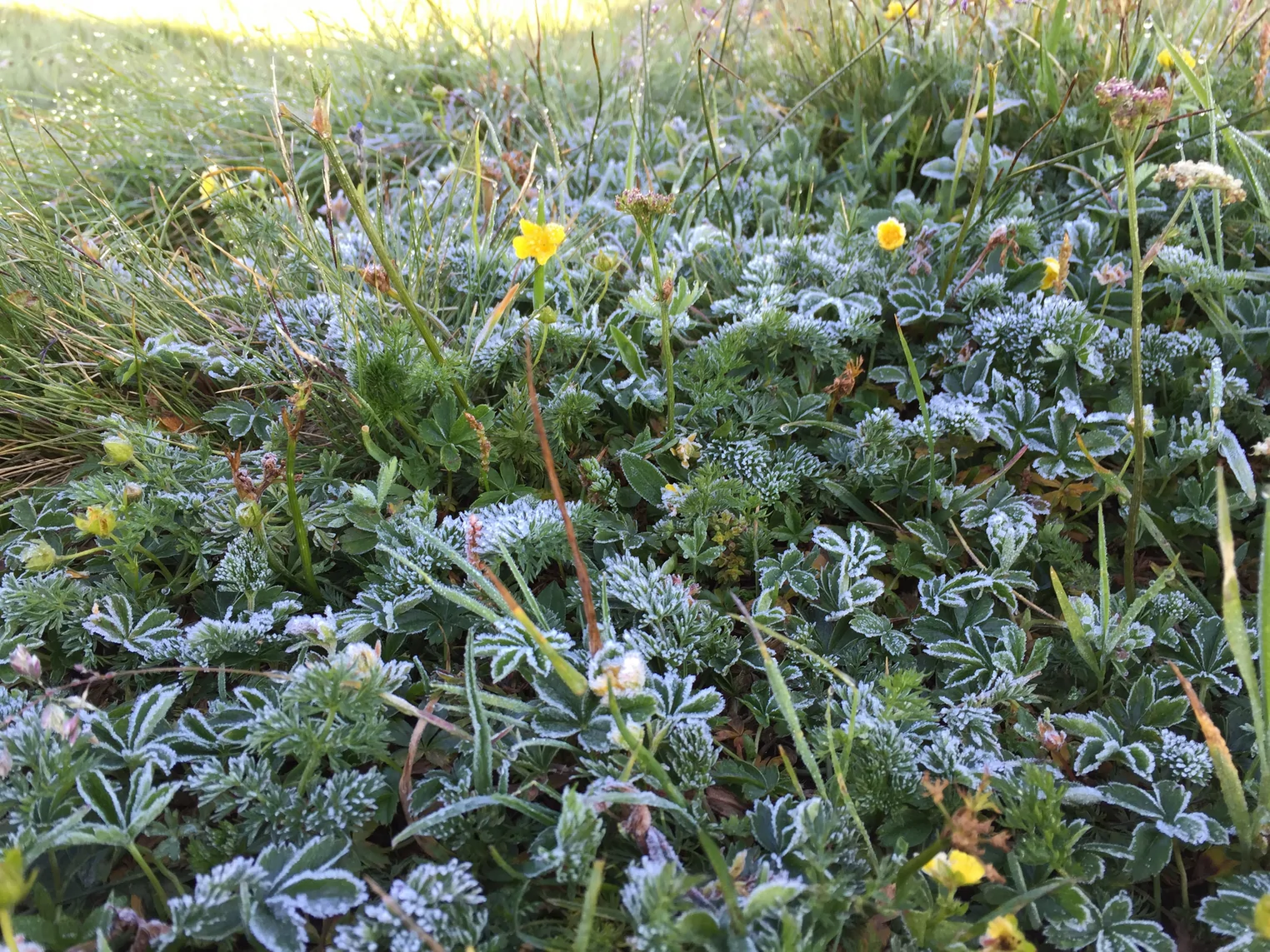
Formation of hoarfrost on top canopy structures exposed to a clear sky. Because of the net heat loss these surfaces cool below air temperature. Topography driven shelter reduces a convective heat exchange with air temperature (at 2440 m a.s.l, Furka Pass, Switzerland, 7 August at 6 am, 2016; photo: C. Körner)

During the day, this interaction and the resulting warming of plants is dominated by solar radiation and depends on the direction (aspect) and angle of slopes (steepness). In clear nights, a net radiative heat loss of plant canopies can occur, depending on the topography: in concave patches, shelter will slow wind speed and thus, reduce convective heat transfer (due to high aerodynamic boundary layer resistance). This may cause surface temperatures to drop below air temperature by several K, often apparent by the formation of hoarfrost on top canopy plant structures (Fig. 2). In contrast, on convex terrain (or in tall alpine plant species), convective air flow may diminish the effect of radiative energy loss to the sky and maintain canopy temperature closer to air temperature. Without an insulating snow layer, plant species in concave terrain (e.g., snowbed species) usually face a higher risk of frost damage, given their lower freezing tolerance (Körner 2021; von Büren and Hiltbrunner 2022).

The convex-concave topography difference incurs effects on soil development (quality and depth), and thus affects water storage and sensitivity to periodic drought. These effects also become apparent on rock outcrops or cliffs. While the ongoing climatic change enhanced vascular species richness on mountain summits from the Western to the Eastern Alps, it diminished species richness in the southern, Mediterranean mountains (Pauli et al. 2012; Rosbakh et al. 2024), most likely because of an interaction between warmer and drier summers and topography-driven soil depth reductions on these summits. The topography-related absence of soils on rocks opens space for alpine succulents (e.g., *Sempervivum*, *Sedum*, *Saxifraga* species) with partly employing CAM metabolism (Wagner and Larcher 1981; Codignola et al. 1990).

For two of the most abundant alpine graminoids (*Carex curvula* and *Nardus stricta*) in the European Alps, which co-occur in the alpine terrain over acidic soils, von Büren and Hiltbrunner (2022) found that minimum winter temperatures during a snow-poor winter define the distribution patterns. Microtopography affected snow cover and thereby protected *Nardus* from critical freezing temperatures, while growing season length and soil quality did not contribute to the explanation of distribution pattern of these species (von Büren and Hiltbrunner 2022). The study underscores that the type of temperature obtained is key (a temperature measured close to vital tissues and meristems; see Sect.  4). Mean annual temperature (MAT), although still often reported, is of no use, particularly, when plants are under snow and dormant. And the hierarchy and interrelationships among influential environmental factors clearly matters.

The challenges ahead are identifying the soil and microclimatic patterns that control plant distribution at the < 1 m scale. Can the edaphic patterns be separated from microclimatic ones? For the alpine flora (beyond bedrock chemistry), we expect overarching influences of topography associated snow duration (see section on snow duration) and microhabitat temperatures as primary drivers, with nutrients through water availability (soil moisture) and soil microbiome rather representing co-consequences. If measured at the most relevant soil depth and without a probing bias (see section on plant temperature), temperature has been shown to represent a proxy for plant occurrence (species distribution ranges), both through a gradual influence (growing season means, Scherrer and Körner 2011) as well as through winter extremes, hence, including critical thresholds (von Büren and Hiltbrunner 2022).

### The nunatak climate in the European Alps

**Fig. 3:**
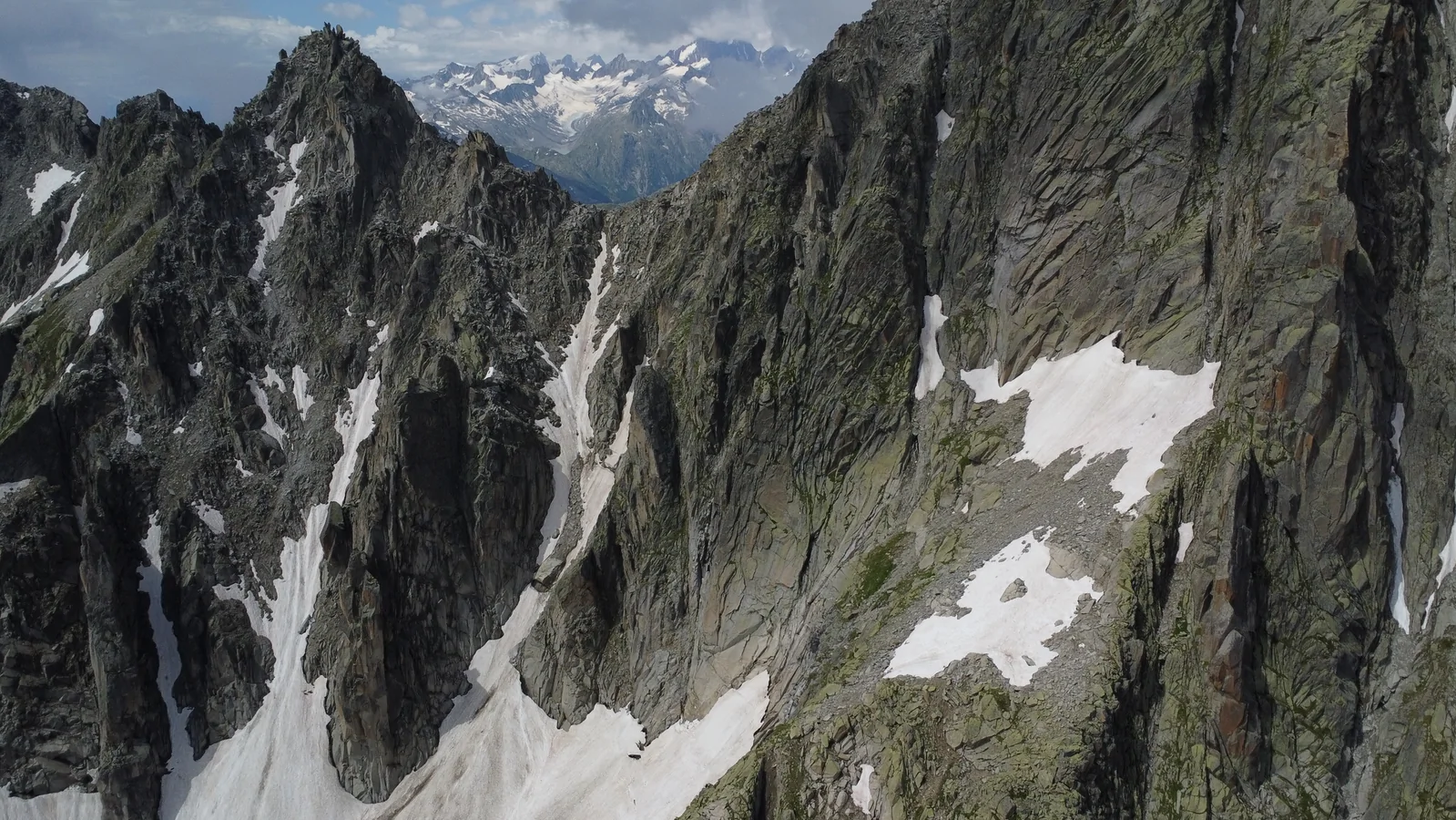
A likely nunatak landscape that might have provided topography-shaped refugia during the glacial era for the current alpine flora (summits: right‒Gross Furkahorn 3168 m a.s.l., left‒Sidelenhorn 3217 m a.s.l. (summit not visible, central Swiss Alps). Finsteraarhorn (cloud covered summit, 4274 m a.s.l.) and Finsteraar glacier in the background (drone-photo: L. Holzer)

During glacial periods, topography, in this case steep and high mountain rocks protruding through the ice shield, created microhabitats that potentially permitted a suite of high alpine taxa to persist through the glacial era, and then descended by the end of the glacial period, contributing to the post-glacial high alpine flora (Fig. 3). This idea was born in the late 19th century and has been widely discussed since then (e.g., Merxmüller and Pölt 1954; Gugerli and Holderegger 2001; Birks 2019; Rota et al. 2024; Rosa et al. 2025). However, the actual life conditions that could support such nunatak plant life has hardly been explored (e.g., Körner 2011).

**Fig. 4:**
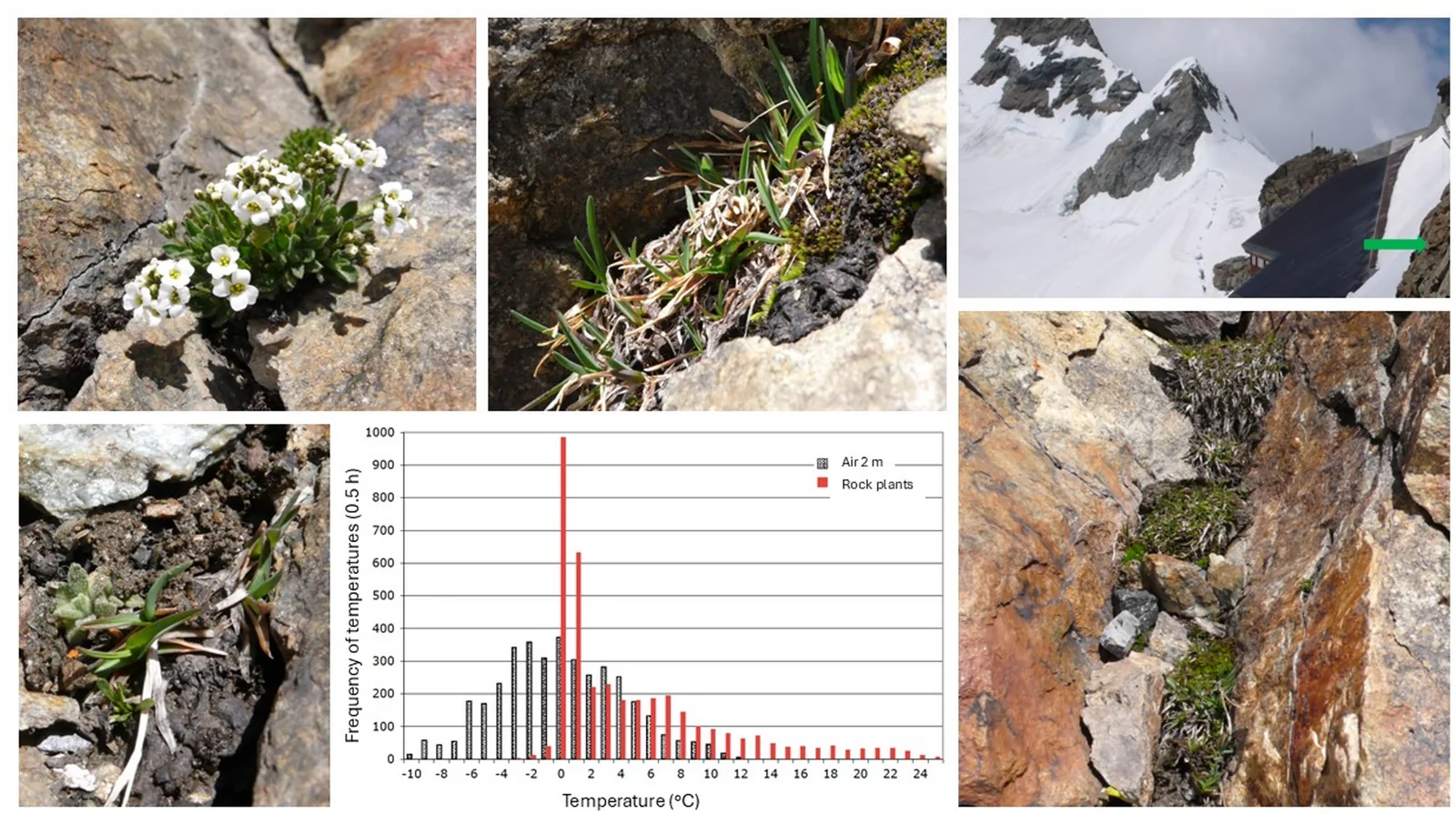
Temperatures recorded at c. 3 cm depth (0.5 h interval) under rock crevice plants (S-exposed, see green arrow) at Jungfraujoch in the Swiss Alps and associated 2 m air temperature at 3470 m a.s.l. for 6 July–21 September 2010. *Draba siliquosa* in full bloom and leaves of *Poa* cf. *supina*

One of the earliest attempts at assessing temperature in rock cliffs above the glacial terrain was that by Mahringer (1964) next to the Sonnblick observatory in the Austrian Alps at 3100 m a.s.l. In addition to rock temperatures, he fortunately included ‘soil’ temperatures at 2 cm and 10 cm depth under bare ground nearby, both measured year-round and compared with standardized air temperature readings. During July-August 1961–1963, he recorded monthly mean maxima of 4.7 °C (abs. max 11 °C) for air, 11.9 °C (abs. max 28 °C) for 2 cm soil and 17–19 °C in 2 cm rock depth (abs. max of 29 and 33 °C) for flat vs. inclined at 30° S-exposure. During July and August 1962, 31 days exhibited below zero air temperatures, but only five days at 2 cm soil depth did so. The mean diurnal amplitude of temperature for all days was around 4 K in air and 2–11 K in soil, but showed daily differences of 13–20 K on bright days. Obviously, this bare substrate became substantially warmer than air temperature, even when averaged across all weather conditions, but particularly so under bright conditions. On bright days, the heat flux into rocks was almost twice as fast than in soil, illustrating that plants living in narrow rock crevices, profit from the heat absorbed (and stored) by the surrounding rock. Using small temperature loggers, permitted a quantitative assessment of the life conditions for rock plants at Jungfraujoch in the Swiss Alps at 3460 m a.s.l. (Fig. 4).

The temperature measured immediately under plants growing in a nunatak-type habitat was recorded on a cliff, directly above the High Altitude Research Station Jungfraujoch in the Bernese Alps of Switzerland. Several different angiosperm and cryptogam species are growing in crevices on that S-exposed cliff at 3470 m elevation, in addition to the ones shown in Fig. 4: *Ranunculus glacialis*, *Festuca* sp., and species of Caryophyllaceae and Saxifragaceae. A temperature logger (TidbiT v2, Onset Hobo, U.S.) was embedded in the fine-substrate, c. 3 cm under plants in crevices. Reference air temperature was recorded by the same logger type under an open shelter of the adjacent station building, screened against the sky. The Jungfraujoch station is known for its high-altitude record temperatures near the ‘Sphinx’ observatory at 3582 m a.s.l., with the single highest and lowest air temperature recorded between 1933 and 2018 +12.8 °C (19 Aug 2012) and -36.6 °C (6 March 1971), respectively.

The summer temperature recorded at this cliff (including all days from 6 July–21 Sept), irrespective of snow cover) revealed a massive shift towards warmer temperatures where plants grow, and hardly any freezing (T range of -2.4 to +25.8 °C), because bad weather is often accompanied with insulating snow and the rock’s body strongly buffers against short-term cooling of the apical- and root-zone of these plants. The hours above 0 °C or 3 °C for this 78-day-period were 988 h–531 h for air vs. 1840 h–931 h for plant temperature, respectively. Thus, plants had nearly twice as many hours above 0–3 °C than measured in air. Plants experienced a total of 328 h between 10 and 25 °C. The mean temperature for the entire period was 0.5 °C for air, but 5.2 °C for soil temperature under plants. At the 1 km higher location at the Swiss Dom summit, a mean of 2.6 °C (soil temperature) was recorded for all 66 days with at least 1 h per day above 0 °C (Körner 2011). A similar mean in soil temperature of 2.9 °C (but for 10 cm depth) was reported by Dvorský et al. (2015) beneath five vascular plant species at 6150 m a.s.l. in Ladakh (Himalaya).

Attempts at reconstructing the temperature regime during the last glacial maximum (LGM) arrived at a range of mean annual temperatures (MAT) of 5–12 K below current (‘current’ is rarely defined), with different proxies, modelling methods, test regions, and seasons arriving at inconsistent estimates (e.g., Jouvet et al. 2023; Rentier et al. 2025). Biswas et al. (2020a, b), obtained MAT depressions for LGM from luminescence paleotemperature on feldspar in the Western Alps, and Bekaert et al. (2023) reconstructed paleotemperatures from noble gas records in groundwater from an aquifer in France (Paris Basin), both reported a c. 9 K cooling for the LGM. As today, MAT is a rather useless LGM temperature metric for the seasonal life in mountains (Körner and Hiltbrunner 2018) and the LGM cooling differed massively between winter and summer (see below).

A recent synthesis of pollen data and the associated type of vegetation and its thermal affiliation across Europe (though, the studied mires and lakes were all outside the ice sheets of the European Alps) revealed (a) a strong seasonality (winters cooled much more than summers), (b) a strong geographical variation and (c) much less overall cooling in the range of -2 to -7 K, mostly around −3.5 K for the growing season (-8 K for winter), against the air T mean for 1970–2000 as a reference (Davis et al. 2024). These more moderate LGM cooling estimates for summer are corroborated by recent speleothem data (stalagmite isotope signals, E. Hofmeister and D. Fleitmann, Jura mountains, pers. com.) that suggest a 6.5 K cooling during the LGM compared to current. Because of the rapid climate warming in recent years, it makes a big difference what is defined as ‘current’. Air temperature rose by 2 K in the Alps since 1980, but some of these proxies do not cover such a fine temporal resolution.

Assuming a similar lapse rate as today (0.55 K 100 m^−1^), a 9 K difference would correspond to c. 1600 m altitudinal difference in air temperature while a 5 K cooling would amount to c. 900 m difference. For the Jungfraujoch site’s conditions, such a shift would end at either c. 1900–2600 m a.s.l. during the LGM, the first obviously deeply under glacial ice, the latter just above the ice dome.

In a first approximation, a proportional shift of the temperature data distribution under plants recorded at Jungfraujoch by 9 K to the left in Fig. 4, leads to sums of 61 and 381 h for air and plant temperatures above 0 °C, and means for these hours ≥ 0 °C are 1.2 and 5.8 °C, respectively. These hours ≥ 0 °C (for plants) would occur at 44 out of 78 days. Thus, the time window for plant growth or at least persistence at this estimated LGM temperature gets really short in such rock niches at c. 3500 m a.s.l. Despite recurrent hours ≥ 0 °C, the estimated LGM mean plant temperature for all these days of -3.8 °C is much colder than that under the current conditions experienced by *Saxifraga oppositifolia* near the summit of the Dom at 4505 m a.s.l. A 9 K LGM cooling applied to growing seasons suggests a possibility of nunatak plant growth below 3000 m elevation only, provided full south-exposure and shelter. Whereas a 5 K LGM cooling during the growing season could open a nunatak plant world up to 3600 m elevation.

Accounting for the LGM glacial ice thickness, with ice surfaces between 2500 and 3000 m a.s.l. in the European Alps (Ivy-Ochs 2015; Leger et al. 2025), a 9 K cooling permits a relatively small range of elevations suitable for a plant life on nunataks. For a LGM position at 2700 m a.s.l. (4.5 K cooler than those at the Jungfraujoch site), a mean of plant temperatures of 0.74 °C (T range of -6.7 to 21.0 °C) with 766 h ≥ 0 °C and 356 h ≥ 5 °C was estimated, thus conditions that could evidently bear alpine plant life. Far stronger shelter and solar heating effects than observed at the Jungfraujoch would be required for more species to survive at elevations a few hundred meters higher. Hence, all these estimates strongly depend on the LGM cooling during the growing seasons.

It had been assumed that snow rich winters contributed to the advance of glaciers during the last glacial maxima in the European Alps (Spötl et al. 2021; Cartapanis et al. 2025). In potential nunatak refugia, massive snowpack at lower elevations outcrops could render them unsuitable for plant persistence, but could insulate high elevation nunataks during the most hostile periods. On the other hand, cold periods in earth’s history have often been regarded as periods with low precipitation, potentially enhancing the number of hours with solar heating. In general, besides the topography-shaped temperature regime, geology (siliceous vs. calcareous) and position of the nunataks (extent of isolation) have selected for the set of plant species that potentially survived glacial periods on nunataks (Dentant et al. 2024; Rosa et al. 2025).

The challenges here are twofold: we need more confident estimates for LGM air temperatures (with a clearly defined ‘current’ year reference), particularly, for summer periods within potential nunatak regions, and a detailed assessment of the plant temperatures in today’s nunatak landscapes. As the above discussion highlights, a trustworthy reconstruction of nunatak habitats rests entirely on a reconstruction of the actual life conditions and cannot be achieved through meters of elevation or air temperature proxies alone. Temperate mountain summit regions (> 3000 m a.s.l) and current nunatak situations in the high Arctic (Svalbard > 600 m a.s.l., Greenland > 2000 m a.s.l) can serve as ‘living proxies’ for the potential deviation of plant temperatures from air temperatures. The flora and the temperatures at such nunatak-proxy sites could tell us, which part of the alpine flora could have survived the LGM above the ice dome. Our first estimates of the thermal regimes and likely elevations suggest either a rather narrow belt above the LGM ice dome, for a small group of particularly tolerant taxa, when applying the 9 K LGM cooling scenario, but a much wider elevation band and species spectrum with the 5 K LMG cooling scenario for growing season air T. However, such extrapolations rest on the assumption that the ecological niche of the species had been conserved (Randin et al. 2006; Pearman et al. 2008).

Because the steady-state treeline position is globally constrained by a 6 °C seasonal mean air temperature (Körner and Hoch 2023), identifying the periglacial position of treeline forming taxa during the LGM seems to represent a largely under-explored, but promising tool for assessing LGM temperature regimes.

### Topography-driven snow duration and growing season temperatures are hardly related

Snowbeds cover large areas in the Alps (an estimate of c. 1750 km^2^ in the Alps by Choler et al. 2025). Snowbeds are the net-outcome of topography and snow redistribution by strong winds in winter, and therefore imprints in the alpine landscape. They are often described as cold places (e.g., Jones et al. 2001; Hülber et al. 2006), yet, such rating depends on the time window considered (Körner et al. 2022; see below). Overall, there is a rich literature on snowbed ecology, starting with the earliest attempts at quantifying the life conditions in concave (hollows) versus convex (ridges) terrain (Billings and Bliss 1959; Friedel 1961) and a number of more recent works explores the constraints of long snow duration on snowbed vegetation, its biomass production and changes in phenology under climatic warming (e.g., Baptist et al. 2010; Carbognani et al. 2016; Little et al. 2016; Lord et al. 2018; Verrall et al. 2023; Oldfather et al. 2024).

The snowbed environment differs climatically from exposed, uphill alpine environments. During the long snow-covered period, snowbeds are much warmer habitats than convex alpine terrain with its periodically thin or absent snow cover, because of the perfect insulation by snow (Edwards et al. 2007; Choler 2018; Körner et al. 2019; Niittynen et al. 2020; Chap. 5 in Körner 2021). The snowpack confines the growing season to a rather short period and the following snow-free period is usually the warmest of the year. In a now nine-year campaign at four locations, 200–300 m above treeline across the European Alps, snow dynamics across sharp snow duration gradients had been studied together with the associated plant and soil biology responses (Seeber et al. 2021; Körner et al. 2022). These data illustrate that the temperatures during the snow-free period hardly differed between the earliest and latest snow-free patches, provided that the same time window during the growing season was compared, leaving the short length of the growing season as the main constraint for plant growth and development.

**Fig. 5:**
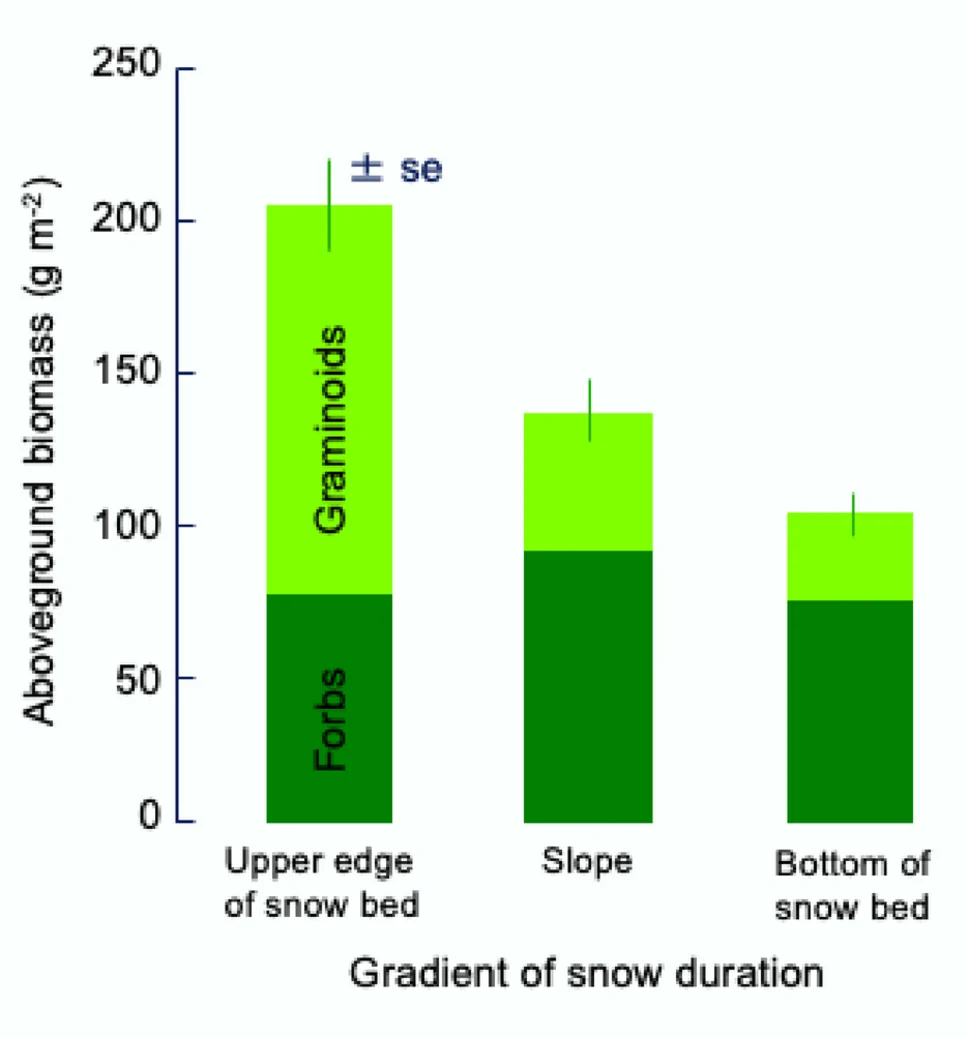
A topography-driven snow duration gradient of 6–7 m that shortens the growing season by 2–3 weeks, causes aboveground biomass to be halved in the bottom of the snowbed compared to the upper edge. Data for 5 snowmelt gradients (*n* = 11‒13 biomass samples per gradient position, means ± SE) close to the Furka Pass (2440 m a.s.l.), Swiss Alps (year 2017, unpublished data)

As the season shortens by 2–3 weeks toward the bottom of the snowbed of a usually c. 10-week growing season for dense alpine grassland, seasonal aboveground biomass production halved, mainly due to the reduction in graminoid species (Fig. 5, for more data see Körner et al. 2022). The peak aboveground biomass varied little across years, reflecting the clonal below-ground structures and the immense root mass (> 85% of total biomass), both, feeding the rapid plant development after snowmelt and buffering biomass produced against weather variability. We suggest that the belowground biomass contributes to this resilience of peak season biomass of snowbed communities. Prior to the current warming period in the European Alps (c. year 1980; see Körner and Hiltbrunner 2024), there were repeatedly years with substantial snowpacks and cold summer temperatures, so that snow in snowbeds as well as in other alpine habitats did not melt and alpine plant species had to survive such “years without summer” (Pfister 1999; Rössler and Brönnimann 2018).

Thus, similar to the nunatak conditions, also in snowbeds there is a critical length of the shortest period that an adapted angiosperm species can cope within an alpine setting. «Coping with» here requires a definition, because it covers survival and growth, both depending on developmental processes, with tissue maturation and amortization representing key criteria. Legacy effects (or the term “extinction debt”, Nomoto and Alexander 2021; Rumpf et al. 2019) could mask the on-going impacts on perennial plants, because different conditions might have been required for the plants to establish, compared to those to persist and to those that enable formation of new plant tissues. The presence of *S. oppositifolia* on the Dom summit, most likely dates back to a single exceptional summer (perhaps 1976, with first observation by climbers 1978). Since then, a few hours above 0 °C during a small number of consecutive days assure its survival (Körner 2011). This species not only reaches the highest summits, but also the most northern latitudes > 83° N (Bay 1992, 2020; reported already by Schwarzenbach in Jones and Richards 1956).

Experimental transplantation in the Western Himalaya at the globally highest elevation limit of vascular plants revealed species-specific survival (species with higher freezing tolerance survived better) during the first three years. However, none of the transplanted species survived at 6100 m a.s.l. (c. 300 m below the highest plant positions) after six years (Dvorský et al. 2016). Authors assumed that an extreme snow event in the last year wiped out the species, hence, not only temperature per se but also topographical shelter and sufficiently long snow-free periods permit plant life at these extreme elevations (Dvorský et al. 2016).

**Fig. 6:**
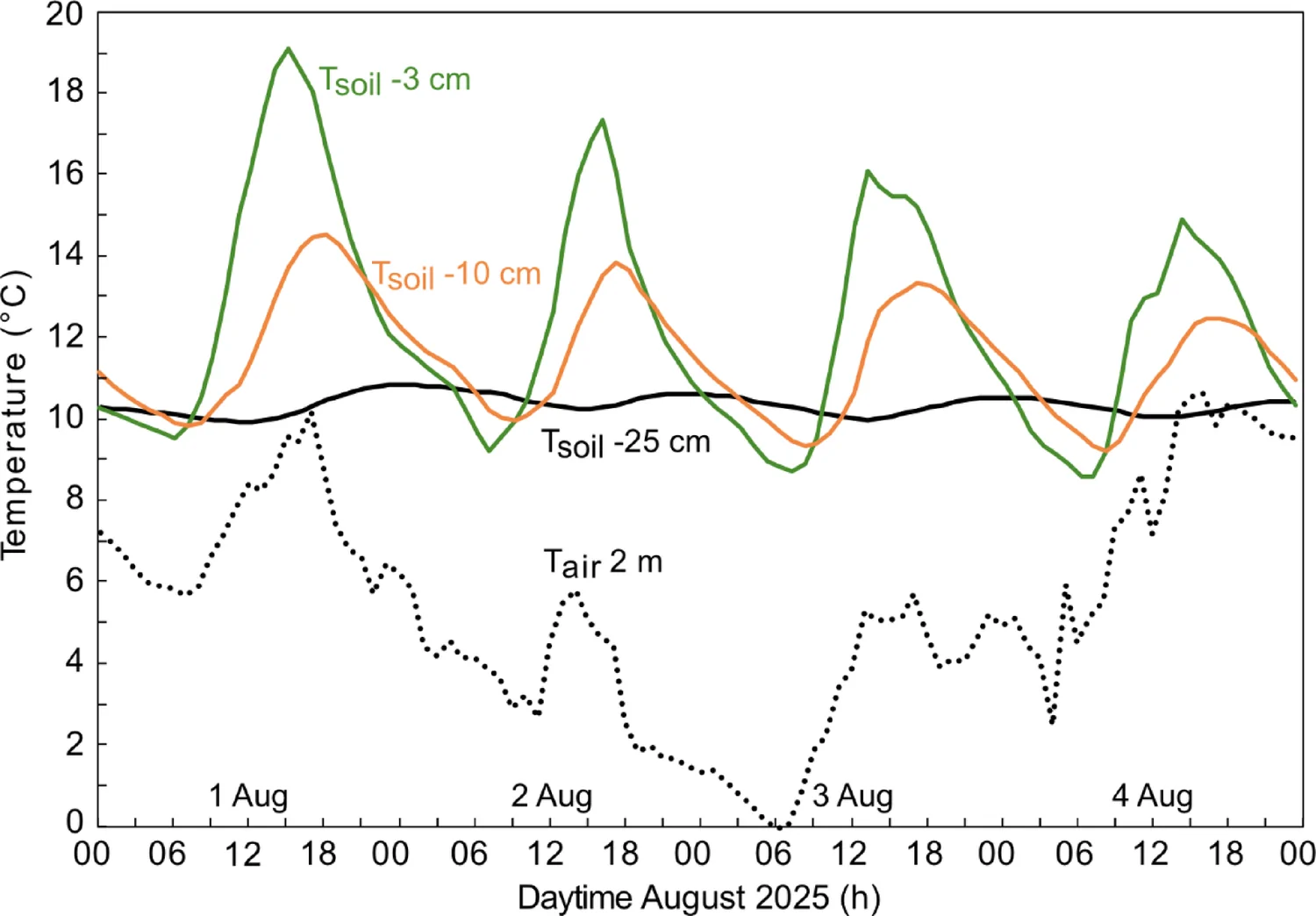
Concurrently recorded temperatures for air at 2 m above ground at the meteorological station ALPFOR (Furka Pass, 2440 m a.s.l.) and in three different soil depths (3, 10, and 25 cm, hourly means) in the immediate vicinity of the meteorological station under alpine grassland during four days in August 2025. Rain fell at the 1st and 2nd of August (daily sum of 4.1 mm and 16 mm, respectively), a cold spell (2nd, 3rd August) lowered air temperature to a minimum of -0.2 °C, but hardly affected the relevant plant conditions in the soil

For plant growth and development (incl. flowering and seed ripening) in alpine (including arctic-alpine) species, the time windows range somewhere between six and eight weeks, depending on how one defines days belonging to the growing season (Molau 1993; Dvorský et al. 2016; Körner et al. 2023). In analogy to a conventional, agricultural 5 °C daily or weekly mean air temperature threshold for growth in crop plants (Körner 2008), this threshold could also be applied to the actual tissue temperatures in alpine plants. Due to the strong decoupling from standard air temperature, a soil temperature of 5 °C at 3–4 cm depth would co-occur with c. 5 K cooler air temperatures (see Fig. 6 as an example). Bürli et al. (2021) reported a mean growing season soil temperature of 4.9 °C (at 10 cm soil depth) or growing season length of 85 days (daily mean ≥ 1 °C), where dense alpine grasslands (40% cover) ends and sparse vegetation begins across European mountains. For sparse vegetation, these authors observed growing season lengths down to 60 days (≥ 1 °C), still above the presumed six to eight weeks limit for many alpine plant species, probably also including autumnal days that do not add to plant growth. The end of the growing season can also be controlled by autonomous (genotypic) senescence, as was shown for the dominant sedge (*Carex curvula*) in an alpine grassland by Möhl et al. (2022).

Because the temperature response of most metabolic processes responsible for growth is non-linear, long-term, arithmetic means are unsuitable to predict growth. What matters is the frequency of suitable hours permitting growth (Fig. 4). It seems that cold adapted crop plants and alpine plants do not differ in their low temperature growth limits: in both, growth slows below 5 °C (asymptotically approaching zero at 0 °C), so that growth during such periods hardly contributes to the seasonal biomass production, but may still be relevant for survival (Körner 2008; Nagelmüller et al. 2016). In strong contrast, the rate of photosynthesis of alpine species studied so far, reaches half of its full capacity at 5 °C leaf temperature, indicating that plant life in cold places is very unlikely to be limited by carbon. The constraints that count are of developmental nature and plant phenology is part of this (Körner 2021).

Populations of plants, including snowbed species that track snowmelt in terms of flowering time, become subdivided into reproductive cohorts that follow the isolines of snowmelt date. While the actual date of release from snow varies a lot across years (> 1 month), the sequence along the recurring snowmelt gradient remains largely conserved. As a consequence, one would expect reproductive guilds that are isolated from each other by their opportunistic flowering phenology. Whether such phenotypic patterns translate into ecotypic (genetic) patterns has rarely been resolved (e.g., Hirao and Kudo 2004; Cortés et al. 2014; Sedlacek et al. 2016). A common-garden experiment with *Carex curvula* clones from earliest and latest snowmelt positions, close to the Furka pass (*n* = 10 for each, unpublished data), however, revealed no genotypic differentiation. The clones kept tracking the snowmelt date in the common garden, hence, flowered jointly 7–10 days after snowmelt. Soil temperatures (at 3–4 cm depth) and calculated thermal sums during phenological observations revealed a strong temperature dependency for the opportunistic flowering behaviour of this species (Vorkauf et al. 2021). Less opportunistic species may either require a certain thermal sum or photoperiod, or a combination of both to start flowering (Keller and Körner 2003; Hülber et al. 2010).

In contrast to the *Carex* example, the widespread alpine *Geum montanum* commonly flowers c. 3 weeks after snowmelt in one blooming event across individuals. In the case of extremely early snowmelt, flowering spreads over 2–3 weeks with a full spectrum from green buds to fruiting within the same 1-m^2^ plot (Fig. 17.15 in Körner 2021). Carbognani et al. (2016) suggested that warmth (later release from snow) enhances flowering synchrony. Vorkauf et al. (2021) specified a gradual photoperiod influence for this species. When *Geum* is released at an early date at which it is sensitive to photoperiod, it requires more thermal energy until flowering. The genotypic (provenances-specific) phenology seems to be less plastic in plant species from colder compared to warmer habitats (Gugger et al. 2015; Schmid et al. 2017), a question requiring more research in a snowmelt-context.

Among the issues to be addressed in the future, the question of genetic isolation of snowmelt guilds within species needs to be resolved. Further, an explanation of the diminished success of graminoids compared to dicots in the snowbed’s centre awaits a clarification and so does the limitations of recruitment from seeds in a terrain that receives continuously propagules from the non-snowbed surroundings. A quantification of the soil moisture dynamics in snowbeds (both, due to poor water holding capacity as well as water logging effects) is wanted. And at the microscale, evidencing that clonal expansion looks promising for identifying expanding snowbed taxa. Assessing the potentially selective role of freezing tolerance in the absence of snow could contribute to a functional understanding of species range limits, “currently” and even more so in a further warming climate.

### How to document topography effects on alpine plant temperature

Here, we summarize a few key criteria for obtaining reliable, ground truth temperature data for alpine plants and habitats. For terminology and the many facets of describing the temperature environment of alpine plants, see Körner and Hiltbrunner (2018). For more technical issues such as sensor type and sensor housing, we refer to Maclean et al. (2021). The task here is to raise awareness of the effect of topography and explain how the mere placement of devices affects the temperature readings.

We recommend that alpine plant ecologists would best refrain from measuring air temperature (for technical reasons that are hard to resolve, but also because alpine plants are decoupled from standardized air temperature). Maclean et al. (2021) made it evident that correctly measuring the air temperature surrounding plants or of leaves, would require sensors of the size of thermocouples with delicate mounting problems as alpine plants have usually small leaves. These are commonly not left-alone devices in an alpine environment, but represent the only way to obtain data, such as the classical demonstration of the effects of microtopography and plant stature for the temperature experienced by alpine dwarf shrubs by Cernusca (1976; see Fig. 4.8 in Körner 2021). While assessing aboveground temperatures in and around alpine plants will remain the domain of specialists, alpine plant ecologist might employ infrared thermometry for surface temperature and various approaches to obtain relevant soil temperatures.

Infrared thermometry, either ground-, drone- or satellite-based, approximates momentary surface temperatures based on the emissivity of the observed surfaces (close to emissivity of 1, Scherrer and Körner 2010; see Fig. 1). The crucial issue is the definition of ‘surface’. Unless there is fog, clouds or exceptionally high humidity, the thermal radiation reflects the temperature of whatever surface is seen by the camera. Infrared thermometry has one great advantage and three disadvantages. The advantage is the unique integrating of features across to-be-defined areas (from mm scale to landscape). The disadvantages are (1) except for fixed, recording cameras, the IR signals are usually not continuous for longer periods (for instance, a season), (2) because images include stones, bare soil, wet surfaces, dead materials, snow, top-canopy foliage, etc., the surface temperature represents the cover fraction of these, within an image frame. (3) The most critical tissues and organs in which temperature exerts a decisive role, meristems in particular, are not visible, but commonly a few cm below ground for the majority of alpine plants. Such remote sensing data should therefore be combined with recording devices at meristem level.

Measuring belowground temperatures seems straightforward. With devices as the ones employed for Fig. 4, records at hourly or finer resolution are easily obtained for years and can be related to the plants’ performance on that spot. Most of the precautions required to acquire trustworthy aboveground temperatures (Maclean et al. 2021) can be neglected here. However, there are two aspects that massively influence the data: the depth of the sensor in the soil and the surface conditions above it (Fig. 6, Fig. S1).

**Fig. 7:**
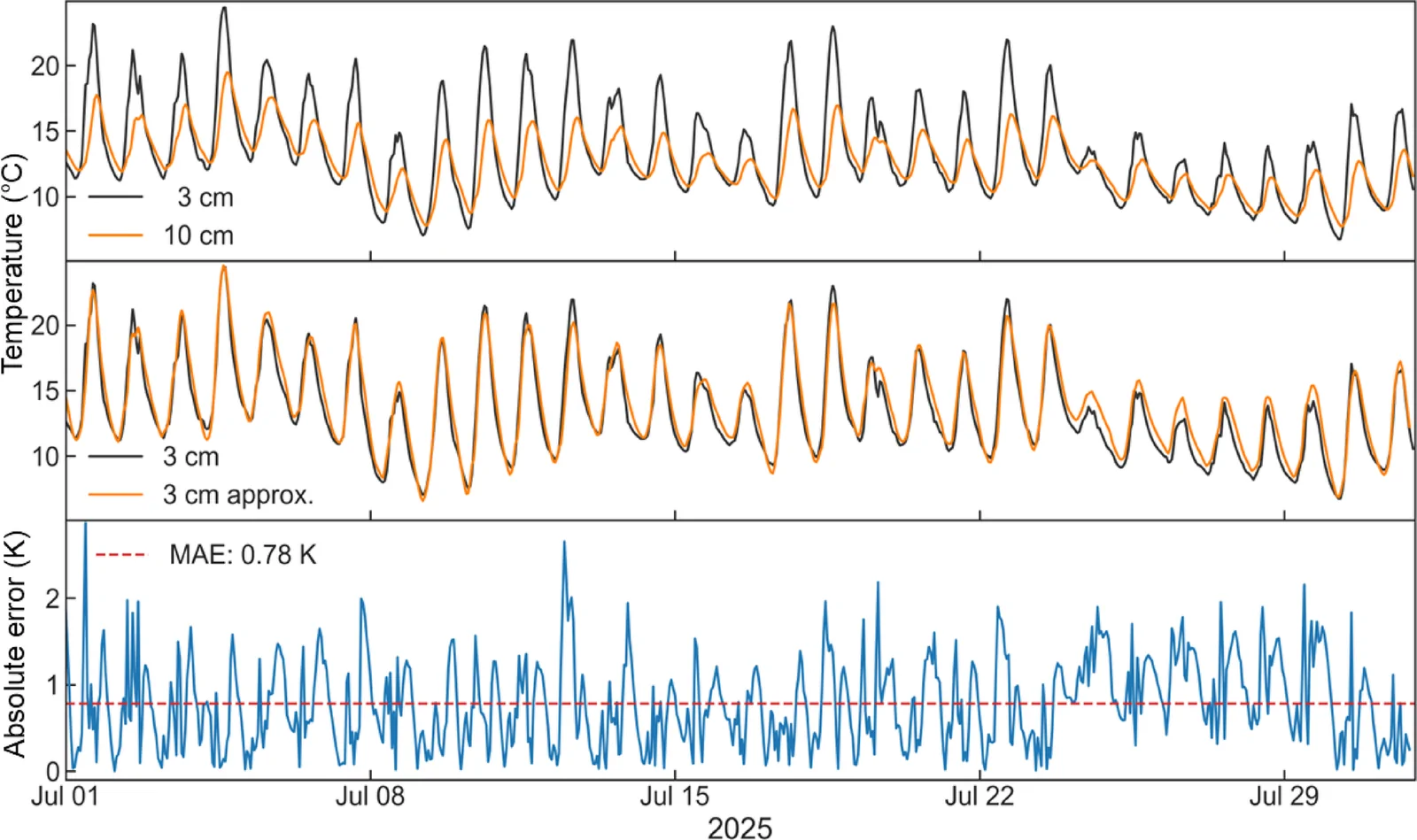
Estimates of temperatures at 3 cm soil depth by T readings at 10 cm soil depth (identical soil type). Uppermost panel shows the original T readings at 3 cm (black) and 10 cm (orange), the middle panel the estimated values at 3 cm from T readings at 10 cm (in orange), overlayed on the measured T at 3 cm (black). The lowest panel displays the absolute errors for each timepoint (in blue, every hour) and the mean absolute error of 0.78 K for the whole period (dashed red line; 1st to 31st of July 2025)

The deeper the probe is buried, the less the signals can be «inflated» by the surface conditions above, but the less relevant the data are for plants and their microbial partners. Burying depth alone causes substantial temperature differences (Figs. 6 and 7), potentially leading to divergent conclusions regarding plant distribution in a microtopography context. For 3–4 cm soil depth, Scherrer and Körner (2010) and von Büren and Hiltbrunner (2022) found a correlation between plant occurrence and microhabitat temperatures. For 10 cm depth, Chytry et al. (2024) found no thermal microtopography effect on plant species distribution, but a strong elevation effect across their 900 1-m^2^ plots over 1700 m elevation gradient on Schrankkogel (Austria). Many of these plots were in the subnival and nival belt with rather sparse ground cover, hence the combination of deeper burial and «uncontrolled» ground cover could have contributed to this divergent outcome.

We suggest a burial of sensors at 3–4 cm soil depth (close to the majority of plant meristems), but with great caution regarding ground cover. Vegetation cover should be representative for the plant and vegetation type studied. A sensor under a closed alpine grassland cover at 2440 m a.s.l. compared to a sensor in 15 cm horizonal distance only, but under a small gap in the plant cover showed 4 K warmer temperatures during bright days, but 2 K cooler ones during clear nights (Fig. S1). A burying depth of 3–4 cm soil depth also requires small sensors for precise placement and to minimize the disturbance of vegetation and soil.

It is well known that the heat flux (though, we focus on temperature here) into the soil is characterised as a damped wave that propagates from the surface (heat), following a diurnal cycle, downwards into the soil (El-Din 1999; Moene and van Dam 2014). With increasing soil depth, the amplitude between minimum and maximum temperature is dampened and the diurnal cycle is shifted, causing the maximum to occur later, the deeper in the soil. In a depth of 25 cm and deeper, the daily maximum value shifts to the second half of the night (next day). This also highlights that temperature records across different soil depths cannot directly be compared. Surface conditions (topography, vegetation cover), soil moisture, soil type and soil homogeneity (e.g., presence of stones) additionally influence the propagation of the heat. While more complex models use Fourier series to describe the heat flux as the sum of multiple sine functions, this is usually beyond practical application and generalisation.

As an example of the depth effect, and to illustrate the decoupling from standard air temperature, we show temperature records in three soil depths in an alpine setting for four days in early August 2025 (Fig. 6). During the four days, air temperature almost never overlapped with the temperatures in the soil, underpinning that air temperature does not reflect the conditions near the plants and the overall much warmer conditions in the soil and for the plants. The daily means (across 4 days) for air were 5.4 °C but 12.3, 11.5, 10.4 °C at 3, 10 and 25 cm soil depth, respectively. Daily maxima varied between 2.5 and 10.8 °C for air, 14.9–19.2 °C at 3 cm, 12–14.6 °C at 10 cm, and stayed nearly constant between 10.3 and 10.8 °C in 25 cm soil depth.

In practice, soil temperatures are often available from one depth only (e.g., in 10 cm), but it would be helpful to have estimates closer to the plant meristems (e.g., 3–4 cm) and to relate these to aboveground plant responses (e.g., leaf expansion rates). Very similar soil conditions at different soil depth represent a pre-requirement for such a comparison. As a first assumption, both, the dampening of the amplitude and the diurnal shift of the T oscillation were taken into account (we use the term oscillation for a fixed point, and wave, when it propagates). Temperatures for 3 cm soil depth were modelled by taking the temperature readings at 10 cm and compared these estimates with real T readings at 3 cm (loggers adjacent to each other), to quantify the errors of the estimates (Fig. 7). As the T oscillation is not constant over time, the rolling daily mean of the 10 cm position was taken. The amplitude at 3 cm was nearly twice (ca. 5 K) of that at 10 cm (ca. 2.5 K). At 3 cm depth, daily minimum and maximum temperature was reached at around 6:00 and 15:00 respectively, whereas at 10 cm, these peaks occurred 2 h later (shift of 2 h, Fig. 7). Compared with the measured temperatures, the errors of these estimates varied between 0 and 20% (absolute percentage error for each timepoint) and with a mean temperature error of 0.8 K for the whole 1.5-month period. Such estimates obviously deviate much more when soil conditions differ between the measured soil depth and the estimated soil depth. By using a nearby 10 cm logger, the mean absolute temperature error was 1.5 K, thus, more than doubled (data not shown).

#### Caution with popular instruments

Given the importance of microclimate, it has become increasingly popular to employ so-called microclimate data loggers TMS (TOMST, Prague, Czech Rep.) which offer all-in-one records for above- and belowground life conditions. As attractive such an approach might be to field biologists, the aboveground data may be quite erroneous. With an overall logger length of 29 cm (half below-, half aboveground), the device promises a c. 15 cm air, a close to zero cm soil surface and a c. 7 cm depth soil temperature, plus a 0–14 cm depth soil moisture mean, at a relatively low price and with long battery life. The soil temperature (7 cm soil depth) and the soil moisture generate reliable readings. However, we caution against using the aboveground data in open terrain and vegetation, because the plastic parts warm up dramatically and unpredictably in the sun. While the available two- and threefold screens minimize over-heating from the top, the vertical plastic tube, hosting the two above-ground temperature sensors, heats up in the sun and the heat is apparently conducted to the sensors, causing 8–12 K excess temperature for ‘air’, compared to the temperature readings by a professional device, also 15 cm above the ground. The supposed surface temperature sensor measures up to 20 K above air (Fig. S2).

One may argue that bare soil may reach similar temperatures as a piece of plastic nearby, but variable shade in the canopy causes the records to run out of control, hindering any offset or standard procedure to “correct” these records during day-hours. So, we strongly recommend to use the aboveground data under full shade (e.g., in a closed forest), during nights or under snow only, but ensure that bright weather data are not included in longer-term means or maxima for exposed sites. Under bright conditions the two upper sensors reflect the interaction of solar radiation with a piece of plastic, as also shown in Fig. 1.

We notice a massive bias toward modelling alpine plant distribution from inadequate data sources such as elevation or air temperature or unreliable proxies for microclimate. Hence, future research should best go in three directions: (a) providing a ground-truth variance term to air temperature based projections as developed by Scherrer and Körner (2010) for typical alpine landscapes in the European Alps and in the high Arctic, (b) modelling land surface temperatures from physical principles at very small scales (e.g., Rankinen et al. 2004 for winter; Pape et al. 2009 for summer), and (c) assess more ground truth environmental data that come closest to plant species requirements (such as the examples summarized in Körner 2021 and those by Choler 2018). This requires appropriate sensor placement (depth, spatial replication, ground cover), with consequences as discussed above.

## Conclusions

Topography shapes the environment alpine plants experience. By small plant size and the various effects of topography we describe, alpine plants decouple themselves markedly from the ambient environment. Jointly, topography creates mosaics of soil conditions with variable moisture and nutrient regimes, all selecting for different species.

These habitat mosaics cause the flora to segregate into patches of life conditions, with temperatures that may periodically differ by more than what would correspond to a several hundred meters difference in free atmosphere altitude. We illustrate the central role of aerodynamics and solar radiation in interaction with topography. Topography also affects the degree of radiative cooling in clear nights.

There are good reasons to assume that topography effects were responsible for a part of the alpine flora to survive the glacial periods on nunataks. We demonstrate that today’s climate at the 3500 m Jungfraujoch site offers a reasonable chance of survival for a handful of alpine plant species. An estimated 9 K colder climate during the LGM would clearly shift the mean of plant temperatures under such topographically favourable conditions at Jungfraujoch beyond the physiological range as well as underneath the ice dome (at c. 1900 m). A 5 K LGM cooling would keep a much larger nunatak terrain open for plant life, presumably up to 3000 m or even beyond. Importantly, it is the consecutive number of warm, non-freezing hours that matters. As a result of these warm hours, liquid water might become available and plant physiological processes could set in, decisive for plant’s survival in this world of ice. Nonetheless, these microtopography and microclimate effects could not fully compensate a 9 K colder climate, but could retain vital niches above 3000 m during the LGM with a 5 K cooling estimate during growing seasons. As we discussed, the currently available estimations of growing season temperatures during the LGM are inadequate to sort out the life conditions on nunataks. Plant survival during the LGM was most likely restricted to substantially lower elevations than current highest vascular plant occurrences, and to most robust species of the today’s alpine flora. However, which plant traits and/or physiological properties contribute to such a robustness have not yet been resolved. This relates to the central question in plant ecology, why plants are growing where they do (see epilogue in Körner 2021). Thus, a fruitful research approach should combine spatial occurrence patterns with microclimatology and theory-based physiological hypothesis testing (e.g., Niittynen et al. 2020; von Büren and Hiltbrunner 2022).

The potential micro-evolutionary role of snowmelt patterns, separating reproductive guilds, is another field that awaits further detailed exploration, because it holds promise for divergent phenology responses of alpine plants as climatic warming proceeds. Given the nowadays simple-to-use devices for field ecologists to collect ground truth environmental data (temperature, moisture, etc.) across varied topographies, we offer some guidance toward avoiding common pitfalls of probe placement and analyses. We advise against aboveground meteorological data collection in alpine settings outside of professional installations. We recommend the dominant meristem level at shallower soil depth for temperature records instead. The structure and density of the plant cover and microtopography are important factors that determine the conditions for alpine plant life, both above and below the ground.

## Electronic Supplementary Material

Below is the link to the electrdonic supplementary material.

Supplementary file 1

Supplementary file 2

Supplementary file 3

## Data Availability

No datasets were generated or analysed during the current study.
