# Supplementary material for: Alpine life is shaped by topography
Source: Alp Bot. 2026 Jun 16;136(2):171–87. doi: 10.1007/s00035-026-00360-8 (PMC13695592; doi:10.1007/s00035-026-00360-8)
Supplement: Supplementary file 1 [file 35_2026_360_MOESM3_ESM.docx]

Supplementary Material to

Alpine life is shaped by topography

by Christian Körner and Erika Hiltbrunner

Fig. S1 Vegetation cover immediately above a T probe (TidbiT v2, Onset Hobo, U.S.) buried in 3-4 cm depth affects both maxima and minima. T readings (all in 1-min measuring resolution) for 8 days (in August 2025 (21^st^ to 28^th^ Aug) in the vicinity of the ALPFOR meteorological station in alpine grassland at 2440 m a.s.l. Yellow arrows hint to the white string attached to the T probes. Reference air temperature (2 m above ground) recorded with the same T sensor in a professional housing (Photos: C. Körner)

Fig. S2 Illustration of overheatig effects of plastic housings of the Tomst logger (TOMST TMS, Czech Rep.), unsuitable to obtain aboveground temperatures under alpine field conditions. (a) Concurrent readings of air temperate 2 m above ground (Meteo station Furka) with the so-called air temperature at 15 cm above ground. (b) Surface temperature of the Tomst logger reveals massive overheating. Tomst temperature at 7 cm soil depth shows similar readings than T logger at 10 cm (TidbiT, Onset) at the same grassland spot, but with slightly lower daily maximum values (Photo: C. Körner)
